# *Mycobacterium tuberculosis* components expressed during chronic infection of the lung contribute to long-term control of pulmonary tuberculosis in mice

**DOI:** 10.1038/npjvaccines.2016.12

**Published:** 2016-09-15

**Authors:** Claudio Counoupas, Rachel Pinto, Gayathri Nagalingam, Grant A Hill-Cawthorne, Carl G Feng, Warwick J Britton, James A Triccas

**Affiliations:** 1 Microbial Pathogenesis and Immunity Group, Department of Infectious Diseases and Immunology, Sydney Medical School, University of Sydney, Sydney, NSW, Australia; 2 Tuberculosis Research Program, Centenary Institute, University of Sydney, Sydney, NSW, Australia; 3 Sydney Medical School and The Marie Bashir Institute for Infectious Diseases and Biosecurity, University of Sydney, Sydney, NSW, Australia; 4 School of Public Health, University of Sydney, Sydney, NSW, Australia; 5 Immunology and Host Defense Group, Department of Infectious Diseases and Immunology, Sydney Medical School, University of Sydney, Sydney, NSW, Australia

## Abstract

Tuberculosis (TB) remains a major cause of mortality and morbidity worldwide, yet current control strategies, including the existing BCG vaccine, have had little impact on disease control. The tubercle bacillus modifies protein expression to adapt to chronic infection of the host, and this can potentially be exploited to develop novel therapeutics. We identified the gene encoding the first step of the *Mycobacterium tuberculosis* sulphur assimilation pathway, *cysD,* as highly induced during chronic infection in the mouse lung, suggesting therapies based on CysD could be used to target infection. Vaccination with the composite vaccine CysVac2, a fusion of CysD and the immunogenic Ag85B of *M. tuberculosis,* resulted in the generation of multifunctional CD4^+^ T cells (interferon (IFN)-γ^+^TNF^+^IL-2^+^IL-17^+^) in the lung both pre- and post-aerosol challenge with *M. tuberculosis*. CysVac2 conferred significant protection against pulmonary *M. tuberculosis* challenge and was particularly effective at controlling late-stage infection, a property not shared by BCG. CysVac2 delivered as a booster following BCG vaccination afforded greater protection against *M. tuberculosis* challenge than BCG alone. The antigenic components of CysVac2 were conserved amongst *M. tuberculosis* strains, and protective efficacy afforded by CysVac2 was observed across varying murine MHC haplotypes. Strikingly, administration of CysVac2 to mice previously infected with *M. tuberculosis* reduced bacterial load and immunopathology in the lung compared with BCG-vaccinated mice. These results indicate that CysVac2 warrants further investigation to assess its potential to control pulmonary TB in humans.

## Introduction

Tuberculosis (TB) remains a major cause of mortality and morbidity worldwide, with almost 9.6 million new TB cases and 1.5 million deaths per year.^1^ Two billion people are estimated to be latently infected with *Mycobacterium tuberculosis*, representing an enormous reservoir that may reactivate TB later in life. Modelling of TB spread indicates that an effective vaccine, combined with improved diagnosis and treatment, would be necessary to reduce the burden of disease to the 2050 targets set by the World Health Organization.^2^

The only vaccine currently available for TB is *Mycobacterium bovis* Bacille Calmette–Guérin (BCG), which, with almost 3 billion administrations is one of the most widely used vaccine worldwide.^3^ Unfortunately, despite good efficacy in preventing severe forms of TB in infants,^4^ its effect against pulmonary TB in adulthood is variable, ranging from >90% protection or imparting a negative effect, depending on the field trial.^5^ Efforts to develop new TB vaccines have intensified over the last 20 years, with a number of vaccine candidates now in clinical trials.^6^ Strategies to develop new TB vaccines include modification of BCG to improve its capacity to elicit protective immune responses, attenuation of *M. tuberculosis* by deletion of defined virulence factors and the construction of subunit vaccines based on important protective antigens.^6^ However, the most advanced of these candidates, a modified vaccinia virus expressing the *M. tuberculosis* Ag85A protein (MVA85A), did not show improved efficacy compared with BCG in Phase IIb trials in humans.^7^ This suggests that the continual addition of candidates to the current TB vaccine portfolio is required in order to identify the optimal vaccine for TB control in humans.

The choice of antigen to use in new generation vaccines is of critical consideration for their efficacy. Although *M. tuberculosis* expresses a number of antigens considered to be ‘immunodominant’, such as the secreted Ag85B and ESAT-6 proteins,^8^ there is no consensus on the combination of antigens required to confer optimal protection. An important consideration is the capacity of *M. tuberculosis* to exist in a metabolically dormant phase (during the chronic/latent phase of infection), in which the pattern of protein expression differs from that during active infection.^9,10^ Indeed, this may be one limitation of BCG as the vaccine may be rapidly cleared from the host and not enter this ‘dormant phase’, thus limiting the immune response to latency-associated antigens.^11^ Although there has been a focus on secreted antigens of *M. tuberculosis* as vaccine components, on the basis that they are recognised early during infection, many of the latency-associated antigens are intracellular components of metabolic pathways.^12^ Discovery of antigens that can aid clearance of latent *M. tuberculosis*, or be used to treat established infections, would be a major advance in TB vaccine development.

We have previously identified a family of proteins belonging to the *M. tuberculosis* sulphate assimilation pathway (SAP) as stress and host-cell-induced components of the TB bacillus.^13^ Although SAP members are non-secreted, the proteins were recognised during the course of *M. tuberculosis* infection in mice and humans, and were protective against low-dose aerosol challenge in mice.^14^ Importantly, the SAP antigens are highly expressed during hypoxia and starvation, suggesting that they may be induced during the latent phase of the bacilli.^13,14^ In this report, SAP components were identified as highly expressed during chronic *M. tuberculosis* in mice. A fusion protein vaccine incorporating SAP antigens (CysVac2) was protective in pre- and post-exposure models of TB and could boost the protection afforded by prior BCG vaccination. Therefore the CysVac2 vaccine is a potential candidate to protect against TB in humans.

## Results

### Expression of the sulfate adenylyl-transferase of *M. tuberculosis* is strongly induced in the lung during chronic infection in mice

**Figure 1:**
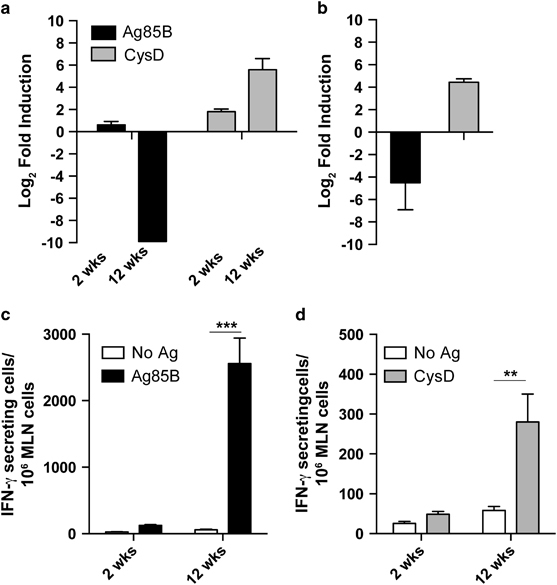
Pulmonary induction of the *M. tuberculosis cysD* gene during chronic infection of mice. C57BL/6 mice (*n*=4–5) were infected with ~100 CFU of *M. tuberculosis* H37Rv by aerosol route. Transcript levels of either *fbpB* and *cysD* were determined by quantitative real-time PCR from *M. tuberculosis* RNA isolated from the lung of mice at 2 and 12 weeks post-infection (**a**) or from DETA-NO-exposed *in vitro M. tuberculosis* cultures (**b**). Data are presented as relative expression of the genes compared with exponential phase *in vitro*-grown *M. tuberculosis* and normalised to the expression of the 16S rRNA gene. The IFN-γ response of MLN cells to Ag85B (**c**) or CysD (**d**) was determined by ELISPOT at 2 and 12 weeks post-infection and compared with unstimulated cells. Data are presented as IFN-γ producing cells per million cells±s.e.m., and are representative of two independent experiments. The significance of differences between groups was determined by two-way analysis of variance (ANOVA) (*P<0.05; **P<0.01).

Sulfur assimilation is critical for *M. tuberculosis* survival and components of the sulfate assimilation pathway are highly expressed under conditions of sulfur limitation and within macrophages, the principal host cell for *M. tuberculosis* during infection.^13,14^ Furthermore, transposon mutagenesis studies showed that genes of this pathway, including *cysD*, are essential for *M. tuberculosis* viability.^15^ The upregulation of *cysD* within host cells, coupled with the requirement for sulfate assimilation during chronic *M. tuberculosis* infection,^16^ suggested that expression of CysD may be modified in the host during chronic *M. tuberculosis* infection. To test this, mice were infected with low-dose aerosol *M. tuberculosis* H37Rv and expression of *cysD* transcript examined at acute and chronic stages of infection. Compared with expression within *M. tuberculosis* broth culture, *cysD* transcript was increased at both acute (2 weeks) and chronic (12 weeks) time points post-infection, with an approximate 6 Log_2_-fold increase at the chronic time point (Figure 1a). The resultant immune recognition of CysD, based on detection of CysD-reactive interferon (IFN)-γ-secreting cells in the mediastinal lymph nodes, was more pronounced at 12 weeks post-infection, reflecting the heightened gene expression at this time point (Figure 1d). *CysD* expression was also compared to that of Ag85B (*fbpB* gene), an early, secreted antigen of *M. tuberculosis*. As previously reported,^17^ *fbpB* transcript was apparent at 2 weeks post-infection but the gene was downregulated at 12 weeks post-infection (Figure 1a). Despite this reduction in *fbpB* transcript, which may be expected to reduce the persistence of Ag85B-specific memory T-cell populations, high levels of Ag85B reactive IFN-γ secreting cells were detected at 12 weeks post-*M. tuberculosis* infection, indicating persistence of the Ag85B response during the chronic phase (Figure 1d). To further define the expression profile of *cysD* and *fbpB*, messenger RNA (mRNA) expression was examined in the presence of the nitric oxide donor DETA-NO, which arrests replication of *M. tuberculosis* in broth culture.^18^ The *cysD* transcript was increased 5 Log_2_-fold by DETA-NO addition, whereas *fbpB* levels decreased by 4 Log_2_-fold (Figure 1b). Therefore, the induction of *cysD* during chronic *M. tuberculosis* infection may be due in part to the arrest of *M. tuberculosis* replication within the host, possibly caused by host NO production in IFN-γ-activated macrophages.^19^

### A CysD-based vaccine affords significant protection against pulmonary *M. tuberculosis*

The ATP sulfurylase enzyme is recognised by the immune system of TB patients, and the two subunits of the complex, CysD (adenylyltransferase) and CysNC (GTPase/APS kinase) confer partial protection against *M. tuberculosis* infection in mice when delivered as DNA vaccines.^14^ Coupled with the heightened CysD expression and immune recognition during chronic infection in mice, we surmised that vaccines incorporating CysD could serve to control both acute and chronic infection with *M. tuberculosis*. To test this, we developed a fusion protein vaccine incorporating CysD and Ag85B, the latter selected because of its strong immune recognition in all stages of infection (Figure 1b) and its demonstrated immunogenicity in vaccinated individuals in human trials.^20^ The fusion protein, termed CysVac2, was expressed in *Escherichia coli* and purified to homogeneity by affinity chromatography by virtue of a N-terminal histidine tag. A protein of the expected size (71 kDa) was visualised in western blot using Ag85B-reactive polyclonal sera (not shown).

**Figure 2:**
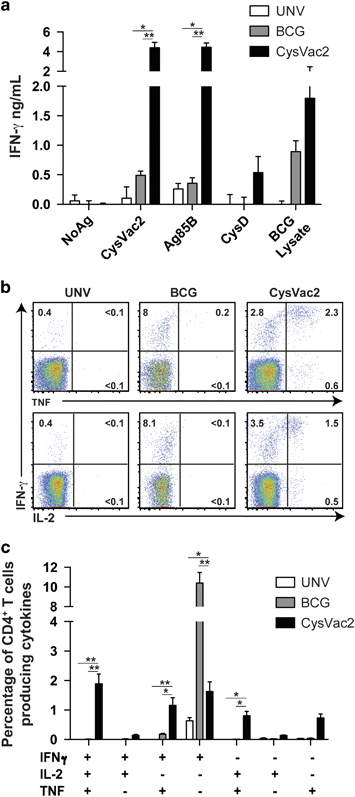
Stimulation of multifunctional CD4^+^ T cells after vaccination with the CysVac2 vaccine. C57BL/6 mice (*n*=4–5 per group) were vaccinated with BCG (s.c., 5×10^5^ CFU) or 3 times s.c. with 3 μg CysVac2 formulated in MPL/DDA. Six weeks after the last vaccination (**a**) the release of IFN-γ from lung cells was determined by ELISA after *in vitro* stimulation with CysVac2, Ag85B, CysD or BCG lysate. (**b**) Representative dot plots of CD4^+^ lung T cells restimulated *ex vivo* with CysVac2 in the presence of brefeldin A and detection of intracellular IFN-γ, IL-2 or TNF. (**c**) The frequency of CysVac2-specific multifunctional CD4^+^ T cells in the lung was also determined. The significance of differences between the groups was determined by one- or two-way analysis of variance (ANOVA) (*P<0.05, **P<0.01). Data are representative of two independent experiments.

In order to assess the vaccine potential of CysVac2, a first series of experiments defined the recognition of the fusion protein components when delivered to mice (formulated in MPL/DDA). In the lung of CysVac2 vaccinated mice, IFN-γ secretion was evident upon restimulation with CysVac2 or Ag85B (Figure 2a). The response to the CysD component was lower than that observed with Ag85B (Figure 2a), but still higher than that observed in the unvaccinated or BCG-vaccinated groups, mirroring the previously reported immunodominance of Ag85B when fused to other antigens.^21^ Minimal responses to either Ag85B or CysD were observed after vaccination with BCG, however, recall with BCG lysate did induce IFN-γ production from lung cells (Figure 2a). CysVac2 vaccination also induced a high percentage of polyfunctional CD4^+^ T cells simultaneously expressing IFN-γ, IL-2 and tumour necrosis factor (TNF), as well as double-positive IFN-γ^+^TNF^+^ and IL-2^+^TNF^+^ CD4^+^ T cells in the lung (Figure 2b). BCG vaccination, however, induced mostly single-positive CD4^+^ IFN-γ^+^ T cells.

**Figure 3:**
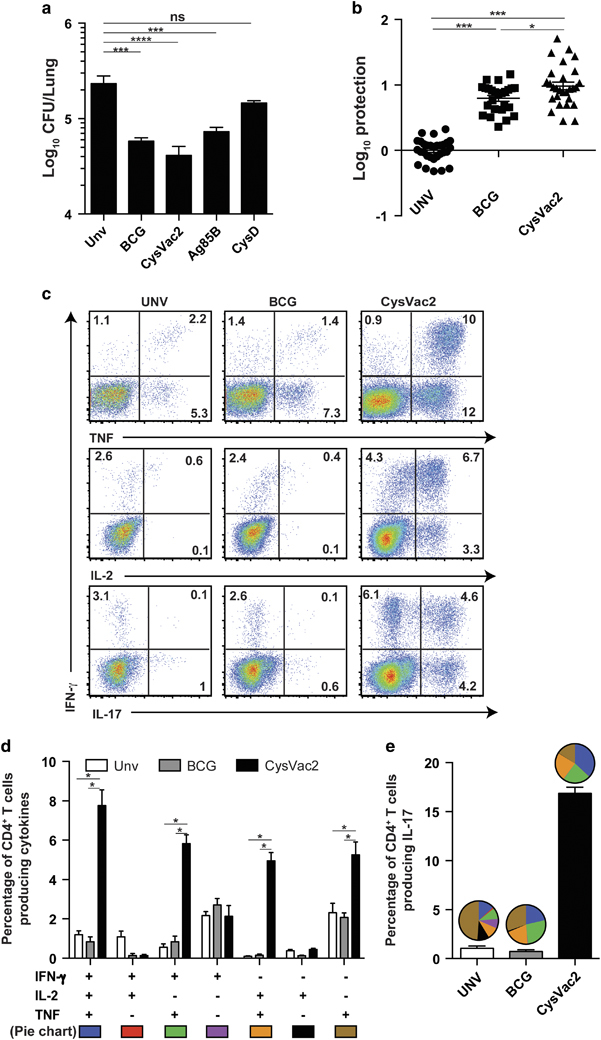
CysVac2-induced protection against pulmonary *M. tuberculosis* infection correlates with the generation of multifunctional CD4^+^ T cells. C57BL/6 mice (*n*=5) were vaccinated with BCG (s.c., 5×10^5^ CFU) or three times s.c. with 3 μg CysVac2, Ag85B or CysD formulated in MPL/DDA. Twelve weeks after the first vaccination, the mice were challenged with ~100 CFU of *M. tuberculosis* by aerosol route and the bacterial load assessed in the lung assessed 4 weeks later (**a**). The data are presented as Log_10_ of the mean of CFU±s.e.m. (**b**) The protective efficacy of CysVac2 was assessed across five individual experiments and a meta-analysis of Log_10_ protection of vaccinated compared with unvaccinated mice shown. (**c**) Representative dot plots of CD4^+^ lung T cells restimulated *ex vivo* with CysVac2 in the presence of brefeldin A and detection of intracellular IFN-γ, IL-2 or TNF. (**d**) The frequency of CysVac2-specific multifunctional CD4^+^ T cells in the lung was also determined. (**e**) The frequency of CysVac2-specific CD4^+^ T cells producing IL-17 in the lung. The proportion of CD4^+^ IL-17^+^ T cells expressing combinations of IFN-γ, IL-2 and/or TNF is shown in the pie chart (see **d** for cytokine subsets). The significance of differences between the groups was determined by one- or two-way analysis of variance (ANOVA) (*P<0.05, **P<0.01; ***P<0.001; NS, not significant). Data are representative of at least two independent experiments.

Vaccination of mice with CysVac2 and subsequent aerosol challenge with *M. tuberculosis* resulted in a significant reduction of pulmonary bacterial load at 4 weeks post-challenge compared with unvaccinated mice (Figure 3a). This response was similar to that afforded by BCG and greater than the protective effect of either Ag85B or CysD alone, demonstrating that maximal protective effect was afforded by the combined fusion protein vaccine. To determine the consistency of protection afforded by CsyVac2, the results of five independent protection experiments were combined, with data normalised to represent the Log_10_ difference in bacterial load of vaccinated mice compared with the mean of non-vaccinated mice in each experiment. Both BCG and CysVac2 provided a statistically significant reduction in *M. tuberculosis* load in the lung (0.79 and 0.98 Log_10_ CFU reduction, respectively, Figure 3b) as well as in the spleen (1.1 and 0.88 Log_10_ CFU reduction, respectively; data not shown). In the lung the protective effect afforded by CysVac2 was greater than that afforded by the BCG vaccine (Figure 3b), indicating the vaccine was highly effective at controlling pulmonary TB in this model.

The quality of the vaccine-specific CD4^+^ T-cell response was evaluated 4 weeks after challenge in the lungs of vaccinated mice. In CysVac2 immunised mice the vaccine-specific responses were dominated by IFN-γ^+^IL-2^+^TNF^+^, double-positive IL-2^+^TNF^+^, and IFN-γ^+^TNF^+^ CD4^+^ T cells subsets (Figure 3c,d). In BCG vaccinated mice, the CysVac2-specific CD4^+^ T-cell response of mice was mainly composed of single-cytokines producing cells and was not significantly greater than that seen in unvaccinated animals (Figure 3c,d). A large proportion of lung CD4^+^ T cells from CysVac2 vaccinated mice produced IL-17, with a high degree of polyfunctionality compared with the correspondent IL-17-producing CD4^+^ T cells from the unvaccinated and BCG groups (Figure 3e). To evaluate the recall response to a broader selection of *M. tuberculosis* antigens, the generation of polyfunctional CD4^+^ T cells in response to *M. tuberculosis* culture filtrate protein (CFP) was examined. Similar to that observed with CysVac2 restimulation, CFP induced a high level of IFN-γ^+^IL-2^+^TNF^+^ CD4+ T cells as well as CD4^+^ T cells producing IL-17 (Supplementary Figure 2a,b). Therefore, CysVac2-vaccinated mice demonstrated a high number of CFP- and CysVac2-specific CD4^+^ T cells with the capacity to secrete multiple cytokines, whereas the frequency of vaccine-specific cytokine-secreting CD4^+^ T cells was low and of a similar frequency between the BCG vaccinated and unvaccinated group.

### CysVac2 can boost the protective effect of BCG against pulmonary TB

**Figure 4:**
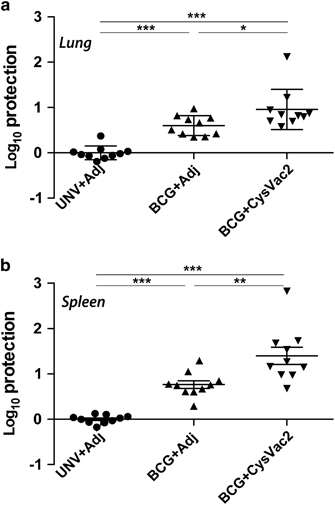
Boosting of BCG protective immunity by CysVac2. C57BL/6 mice (*n*=5) were vaccinated with BCG s.c. or left unvaccinated and 12 weeks later boosted s.c. with 3 doses of CysVac2/MPL-DDA or with MPL-DDA alone. Six weeks after last boost, mice were challenged with ~100 CFU of *M. tuberculosis* by aerosol route and after 4 weeks the bacterial load enumerated in the lung (**a**) and spleen (**b**). Data are combined from two experiments and presented as the Log_10_ CFU difference for each individual mouse compared with the mean of unvaccinated animals. The significance of differences between groups was determined by one-way analysis of variance (ANOVA) (*P<0.05; **P<0.01; ***P<0.001).

BCG protective efficacy wanes over time and homologous boosting with BCG is ineffective in humans,^22^ suggesting vaccines that can boost the BCG-protective immunity would be leading candidates for translation to use in humans. To determine whether CysVac2 could confer this effect, mice were vaccinated with BCG and after 12 weeks were boosted with CysVac2/MPL-DDA or adjuvant alone and then aerosol challenged *M. tuberculosis*. CysVac2 boosting was able to reduce the *M. tuberculosis* bacterial load compared with both the unvaccinated group and BCG-group boosted with adjuvant only, in both the lung (Figure 4a) and the spleen (Figure 4b). Therefore, CysVac2 was able to improve the protective effect afforded by BCG when employed as a boosting vaccine.

### CysVac2 protects against chronic pulmonary infection and reduces bacterial burden when administered post*-M. tuberculosis* exposure

**Figure 5:**
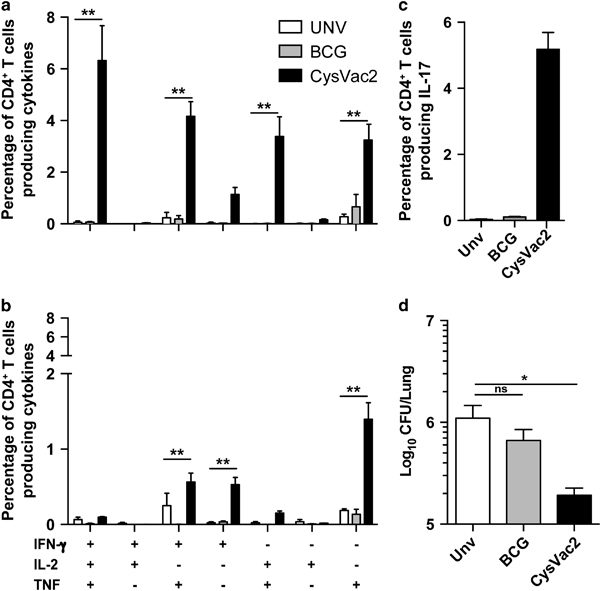
Vaccine-induced protection against chronic lung infection with *M. tuberculosis*. C57BL/6 mice (*n*=5) were vaccinated s.c. with 5×10^5^ CFU BCG or three times s.c. with 3 μg CysVac2. Twelve weeks after the first vaccination, the mice were challenged with approximately 100 CFU of *M. tuberculosis* by aerosol route and 20 weeks the frequency of Ag85B (**a**) or CysD (**b**) specific CD4^+^ T cells producing IFN-γ, IL-2 or TNF or IL-17 (**c**) was determined in lung cells stimulated *ex vivo* with each individual protein in the presence of brefeldin A. *M. tuberculosis* CFU in the lung at 20 weeks post-challenge (**d**). The data are presented as Log_10_ of the mean of CFU±s.e.m. Data are representative of at least two independent experiments. The significance of differences between groups was determined by one- or two-way analysis of variance (ANOVA) (ns=not significant; *P<0.05; **P<0.01).

The marked upregulation of *cysD* expression at later stages of *M. tuberculosis* infection (Figure 1a) suggested that vaccines incorporating this antigen may serve to improve control of chronic infection. In order to address this, the ability of CysVac2 to impact on lung immunity and bacterial burden was examined at 20 weeks post-*M. tuberculosis* challenge. The proportion of Ag85B or CysD-specific cytokine-secreting CD4^+^ T cells in the lung was determined (Figure 5a,b, respectively). CysVac2 vaccination induced heightened generation of multifunctional CD4^+^ T cells subsets. Responses to both antigens were evident at this extended timepoint. However, Ag85B-responding T cells were mostly triple cytokine (IFN-γ^+^IL-2^+^TNF^+^) and double cytokine producers (IFN-γ^+^IL-2^+^ and IL2^+^TNF^+^; Figure 5a), while CysD-responding CD4^+^ T cells were predominately double positive (IFN-γ^+^TNF^+^) and single-positive producers (IFN-γ^+^ and TNF^+^; Figure 5b). A distinct population of CysVac2-specific IL-17-producing CD4^+^ T cells was also observed (Figure 5c). CysVac2 conferred approximately 1 Log_10_ protection compared with unvaccinated mice at this extended time point, demonstrating maintenance of the protective effect of the vaccine in the lung (Figure 5d). Further, the protection afforded by CysVac2 was significantly greater than that imparted by BCG vaccination (Figure 5d). Indeed, BCG failed to reduce *M. tuberculosis* load compared with unvaccinated mice, indicating that BCG-induced protective immunity had waned in this model.

**Figure 6:**
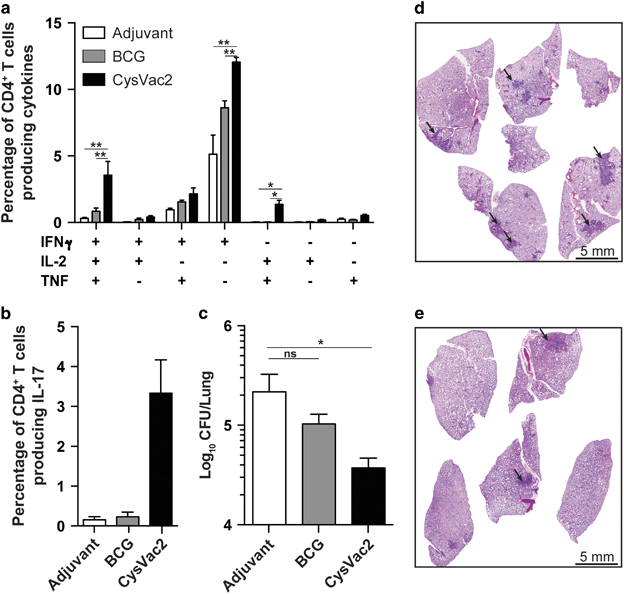
Post-*M. tuberculosis* protective efficacy of CysVac2 delivery. C57BL/6 mice (*n*=5) were infected with ~100 CFU of *M. tuberculosis* by aerosol route and 4 weeks later were vaccinated with BCG or three times s.c. with 3 μg CysVac2 formulated in MPL/DDA. Eight weeks after the last vaccination the frequency of CysVac2-specific CD4^+^ T cells producing IFN-γ, IL-2 or TNF (**a**) or Il-17 (**b**) was determined in lung cells stimulated *ex vivo* with CysVac2 in the presence of brefeldin A. *M. tuberculosis* CFU in the lung at 8 weeks post-vaccination (**c**). The data are presented as Log_10_ of the mean of CFU±s.e.m., and are representative of three independent experiments. The significance of differences between groups was determined by one or two-way analysis of variance (ANOVA) (ns, not significant; *P<0.05; **P<0.01;). Photomicrographs of haematoxylin and eosin (H&E) stain of middle right lobes of unvaccinated mice (**d**) and CysVac2 vaccinated mice (**e**). Arrows indicate granulomas.

When CysVac2 was delivered to mice post-*M. tuberculosis* challenge, the vaccine stimulated the highest proportion IFN-γ^+^IL-2^+^TNF^+^ and CD4^+^IL-2^+^TNF^+^ CD4^+^ T cell compared with adjuvant-treated or BCG-treated mice (Figure 6c) as well as the highest proportion of vaccine-specific CD4^+^ T cells producing IL-17 (Figure 6d). Therapeutic vaccination with CysVac2 significantly decreased pulmonary bacterial load at 4 weeks post-treatment compared with mice vaccinated with adjuvant only (Figure 6a). Vaccination with BCG did not affect the bacterial load in the lung, indicating that BCG is unable to reduce bacterial numbers in this post-exposure setting. Furthermore, histological analysis showed reduced inflammation in the lung with fewer foci of infection in the CysVac2/MPL-DDA-treated group (Figure 6e) compared with the adjuvant only group (Figure 6d). These data indicate that CysVac2 provides sustained protective immunity when used as both a pre-exposure and post-exposure vaccine.

### CysVac2 induces protection across varied MHC haplotypes

**Figure 7:**
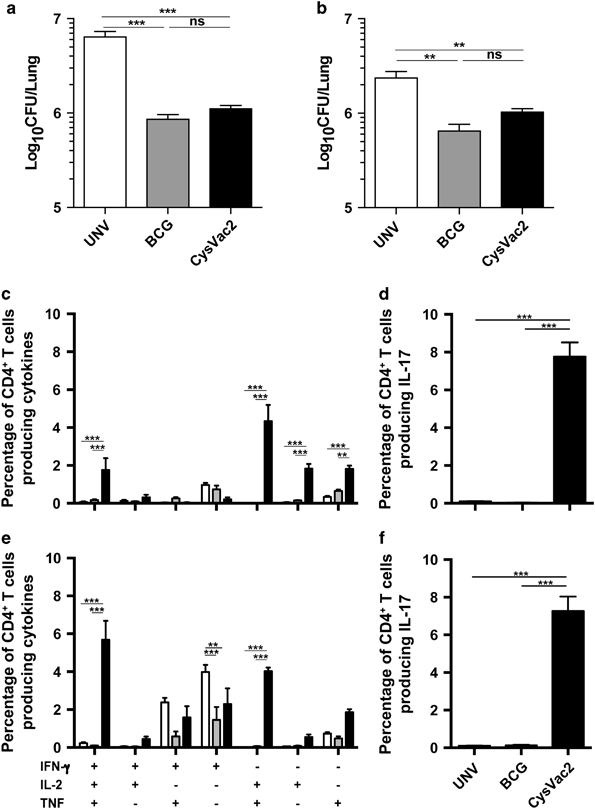
Immunogenicity and protective efficacy of CysVac2 across different MHC haplotypes. BALB/c (*n*=6) or Q(s) mice (*n*=6) were vaccinated s.c. with BCG, or three times with CysVac2 formulated in MPL/DDA. Twelve weeks after the first vaccination, the mice were challenged with ~100 CFU of *M. tuberculosis* by aerosol route and 4 weeks later they were killed and the bacterial load assessed in the lung. The data are presented as Log_10_ of the mean of CFU±s.e.m. (**a**, **b**). The frequency of CysVac2-specific CD4^+^ T cells producing IFN-γ, IL-2 or TNF (**c**, **e**) or IL-17 (**d**, **f**) was determined in lung cells stimulated *ex vivo* with CysVac2 protein in the presence of brefaldin A. The significance of differences between groups was determined by one- or two-way analysis of variance (ANOVA) (NS=not significant; *P<0.05; **P<0.01; ***P<0.0001).

TB vaccine for use in humans should impart protection against infection in populations of differing genetic background. We examined the capacity of CysVac2 to invoke protective immunity in murine strains distinct to C57BL/6 (*H2*^*b*^). BALB/c (*H2*^*d*^) and outbred Q(s) were vaccinated with CysVac2 and protection against aerosol *M. tuberculosis* examined at 4 weeks post-challenge. In both strains, CysVac2 afforded a significant reduction in bacterial load compared with unvaccinated mice, indicating that the vaccine was capable of imparting protective immunity across multiple genetic backgrounds (Figure 7a,b). Protection correlated with the appearance of multifunctional cytokine-secreting CD4^+^ T cells in response to CysVac2 restimulation in the lungs of CysVac2-vaccinated mice (Figure 7c–f); however, differential responses were less evident upon restimulation with CFP (Supplementary Figure 2c–e).

Human T-cell epitopes encoded by mycobacterial antigens, including those of the Ag85 family, are evolutionary conserved.^23^ To examine the diversity in CysD sequence, the sequence variability was examined for all the 1711 *M. tuberculosis* CysD protein annotated sequences available from GenBank to date. This analysis revealed a high degree of CysD conservation, with the majority of amino-acid positions displaying 100% conservation (Supplementary Figure 3a). The same sequence comparison was performed with other *M. tuberculosis* antigens that are components of TB vaccines in clinical trials, including Ag85B.^6^ Conservation for these antigens was high, with limited variability in most amino acid positions (Supplementary Figure 3b), although Ag85A and Ag85B did display greater variation than CysD. These results suggest that CysD is a highly conserved antigen that may impart protective immunity that is independent of the infecting *M. tuberculosis* strain.

## Discussion

During the transition from acute to latent/chronic infection, *M. tuberculosis* undergoes a shift in transcriptomic profile resulting in the expression of ‘latency-associated’ antigens, which facilitate the progression to the dormant state. Indeed, one perceived weakness of the BCG vaccine is its failure to induce immunity to latency-associated antigens, owing to its attenuation and early clearance from the host.^11^ In this study, we observed that the expression of *cysD* was markedly upregulated during chronic stages of infection in the lung of the mice and upon exposure to the nitric oxide donor DETA-NO (Figure 1). These data further strengthen previous findings that demonstrated the induction of *cysD* expression by nutrient starvation and macrophage infection,^13,14^ and also suggest that enhanced sulphur assimilation may be required for adaptation to life within the host. This high level expression of *cysD* during chronic infection resulted in strong immune recognition of CysD in the lung of mice 12 weeks post-*M. tuberculosis* infection (Figure 1), and we exploited this immunogenicity to develop the multistage CysVac2 vaccine. Vaccination with CysVac2 resulted in protection at both acute and chronic stages of *M. tuberculosis* infection, a property not shared by BCG (Figure 5). Importantly, the level of protection observed with CysVac2 either equaled or exceeded that afforded in preclinical studies by TB subunit vaccines in clinical trials.^24–27^ Protection correlated with the induction of CD4^+^ T cells secreting multiple cytokines (IFN-γ, IL-2 and TNF) in CysVac2-vaccinated mice (Figure 2); this subset of cells was reported as a correlate of vaccine-induced, Th1-mediated protection in a *L. major* model of infection^28^ and has been observed in other studies of vaccine-induced protective immunity.^29^ However, despite the apparent link between IFN-γ^+^IL-2^+^TNF^+^ CD4^+^ T cells and protective immunity in animal models, their role in humans is less clear. The most advanced TB vaccine in clinical trials, MVA85A, induces a high frequency of polyfunctional CD4^+^ T cells in humans, however the vaccine was unable to boost BCG-induced protection in a Phase IIb trial.^30^ Further, these cells are detected after BCG vaccination in newborns, but there was no correlation between their frequency and protection against the TB in infants followed for 2 years after vaccination.^31^ A second major CD4^+^ T-cell subset observed after CysVac2 vaccination was composed of cells producing both IL-2 and TNF. In both mice and non-human primates, the IL-2^+^TNF^+^ CD4^+^ subset correlated with improved protection against *M. tuberculosis* infection.^32,33^ In the linear model of differentiation for CD4^+^ Th1 cells, IL-2^+^ CD4^+^ T cell are memory cells with the potential to differentiate into effector IFN-γ-producing cells,^29^ and this cell subset may be of particular importance during chronic infection, since the central memory T-cell pool may become exhausted during a persistent *M. tuberculosis* infection.^34^ Thus, vaccine-induced IL-2^+^TNF^+^ T cells may function as a reservoir for new effector T cells that can replace terminally differentiated and exhausted effector T cells during chronic infection.

CysVac2 vaccination generated a strong Th17 immune response, characterised by the presence of IL-17 secretion by antigen-stimulated T cells (Figure 3). The true role of Th17 CD4^+^ T cells is still somewhat unclear in the context of *M. tuberculosis* infection. Th17 cells correlate with immunopathology during chronic infection;^35^ however, they have also been shown to have a role in the control of *M. tuberculosis* infection, including hypervirulent strains,^36,37^ possibly by facilitating the chemoattraction of Th1 cells into the lung.^38^ DDA is a known potent inducer of Th17 responses and is most likely driving the majority of IL-17 secretion observed after MPL/DDA delivery.^39^ It is possible that a correct balance between Th1 and Th17 response is crucial for optimal protection against *M. tuberculosis* infection. The CAF01 adjuvant (TDB/DDA), which has been used to deliver the H56 subunit vaccine currently in clinical trials, induces a mixed Th1/Th17 response^40^ similar to that seen for CysVac2-MPL/DDA in the current study.

The ability to boost the protection provided by BCG is a key strategy in the development of a more effective vaccine against *M. tuberculosis*. Most TB subunit vaccines in clinical trials are being tested for their efficacy to boost BCG immunity, since it is well established that in adults BCG-induced immunity wanes after ~15 years.^41,42^ In the current study, boosting of BCG-immunised mice with the CysVac2 increased the protection against *M. tuberculosis* challenge provided by BCG alone. A review of 46 independent mouse pre-clinical studies using BCG prime-boosting showed that only a few vaccines could facilitate an improved effect upon BCG, indicating the difficulty in improving upon the protective effect of the current vaccine, at least in well-established animal models.^43^

Post-exposure immunotherapeutic vaccines are considered important components of control strategies to improve control of the global TB epidemic, based on the large reservoir of individuals latently infected with *M. tuberculosis.* Effective post-exposure vaccines administered to persons infected with *M. tuberculosis* would be expected to either reduce the frequency of reactivation or synergise with antibiotic treatment to accelerate bacterial clearance. CysVac2 was able to reduce *M. tuberculosis* bacterial load and pathology when administered as a post-exposure vaccine (Figure 6), which correlates with the strong upregulation of *cysD* during chronic *M. tuberculosis* infection of mice (Figure 1). This effect may not exclusively be due to CysD, as delivery of Ag85B-specific T cells during the chronic course of *M. tuberculosis* infection could improve control of the bacterial burden in mice.^44^ The CysVac2-specific CD4^+^ T cell response in the post-exposure setting was mainly composed of single IFN-γ^+^-expressing cells, a pattern that is associated with terminally differentiated or exhausted CD4^+^ T cells,^29^ and this may be driven by the chronic *M. tuberculosis* infection in this model. Nevertheless, in the lungs of CysVac2-treated mice, triple-positive IFN-γ^+^IL-2^+^TNF^+^ and double-positive IL-2^+^TNF^+^ CD4^+^ T cells were present in greater numbers than in mice vaccinated with BCG or with adjuvant alone. Intriguingly, administration of BCG did not reduce the bacterial load (Figure 6), an effect that has been observed previously, with some studies even demonstrating exacerbation of disease following BCG administration.^45,46^ However, based on this and other studies, delivery of antigens in defined adjuvants does not cause exacerbation of infection.^26,47,48^

CysD is highly conserved amongst *M. tuberculosis* strains (Supplementary Figure 3), confirming the previously reported conservation of important mycobacterial T-cell antigenic epitopes.^23,49^ Conservation of T-cell epitopes is hypothesised to promote T-cell recognition and contribute to the immunopathology associated with disease progression, however such a hypothesis has yet to be proven experimentally. In the case of genes essential for growth, such as *cysD*,^15^ conservation may be a requirement for mycobacterial survival and virulence, particularly if antigenic epitopes span important functional components of the encoded protein (e.g., enzyme active sites). The Ag85B sequence displayed moderately more variability and the *fpbB* gene does contain a mutation in BCG that is predicted to alter the structural integrity/stability of the protein and subsequent immunogenicity.^50^ This may explain in part the paucity of Ag85B-reactive T cells observed after BCG vaccination (Figure 2a). Despite the conservation of CysD and Ag85B sequence among *M. tuberculosis* strains, the epitope ‘repertoire’ encoded by CysVac2 was sufficiently broad to induce a strong immunogenicity and protection against aerosol infection in multiple murine MHC haplotypes (Figure 7), further demonstrating the vaccine potential of CysVac2. The heightened protection afforded in C57BL/6 mice (Figure 3) compared with other murine strains may reflect the strong recognition of Ag85B in this strain.^51^ Therefore, the use of highly protective antigens under selective pressure for survival represents an effective strategy to develop broadly protective TB vaccines.

In summary, this study demonstrates that proteins upregulated during chronic *M. tuberculosis* infection can be exploited to develop vaccines effective in pre-clinical model of pulmonary *M. tuberculosis* challenge. The CysVac2 candidate can protect mice at extended time points after infection and is effective following *M. tuberculosis* exposure, properties not shared by the BCG vaccine. The strong protective efficacy demonstrated by CysVac2 in multiple models of *M. tuberculosis* challenge, coupled with the induction of defined subsets of lung multifunctional CD4^+^ T cell that correlate with protection, warrants further appraisal of the vaccine candidate for the control of pulmonary TB in humans.

## Materials and methods

### Bacterial strains and growth conditions

*E. coli* K-12 and BL21 (DE3) were grown in Luria–Bertani broth or agar (Becton Dickinson (BD), Franklin Lakes, NJ, USA). *M. tuberculosis* H37Rv and BCG strains were grown in Middlebrook 7H9 medium (BD) supplemented with 0.5% glycerol, 0.02% Tyloxapol and 10% albumin-dextrose-catalase or on solid Middlebrook 7H11 medium (BD) supplemented with oleic acid-albumin-dextrose-catalase. All cultures were grown at 37 °C, with or without shaking. Antibiotics were added to media when required, at 25 μg ml^−1^ for kanamycin and 100 μg ml^−1^ for ampicillin. For establishment of non-replicating cultures, 0.1 OD *M. tuberculosis* H37Rv grown in Middlebrook 7H9 was supplemented daily with 50 μmol/l of DETA-NO (Sigma-Aldrich, St Louis, MO, USA) for 7 days.

### Vaccine construction and gene sequence analysis

The plasmid encoding the CysVac2 fusion protein was generated by amplification of the complete coding regions of Ag85B (Rv1886c) from *M. tuberculosis* genomic DNA and cloning into *Nde*I and *Bam*HI sites of pET19b (Novagen, Bayswater, Australia) to generate pET19b-85B. Downstream insertion of the complete *cysD* gene (Rv1285) into the *Bam*HI site of pET19b-85B resulted in pET19b-CysVac2. The pET19b-85B was used for expression of Ag85B alone, while the *cysD* gene was inserted into the *Bam*HI and *Hind*III sites of pET22b(-*pelB*) to construct pET22b-CysD. Plasmids were transformed into *E. coli* strain BCL21 (DE3), selected transformants were grown to an OD_600_ of 0.6 and protein expression induced by 0.3 mmol/l of isopropyl β-D-1-thiogalactopyranoside shaking at 37 °C O/N. The proteins were purified under denaturing conditions using TALON Metal Affinity Resin according to the manufacturer’s instructions (Clontech, Mountain View, CA, USA). Final protein was dialysed in refolding buffer (25 mmol/l HEPES, 0.1 mol/l KCl, 1.5 mmol/l DTT, 5% glycerol, pH 7.5). Purified proteins were visualised by SDS-polyacrylamide gel electrophoresis and detected by Western blot using anti-Ag85B murine polyclonal sera, as previously described.^52^

### mRNA extraction, RT-PCR and real-time PCR

Lungs were collected from euthanized mice and immediately homogenised in 2 ml of Trizol (Life Technologies, Mulgrave, Australia), centrifuged at 3,500 r.p.m. and the pellet resuspended in 1 ml of Trizol. RNA was purified from homogenised lung or *M. tuberculosis* grown in 7H9 media by using the RNeasy kit (Qiagen, Hilden, Germany) and cDNA was synthetised from 1 μg of RNA using Superscript III reverse transcriptase (Life Technologies). Quantitative real-time PCR reactions were performed on cDNA using SYBR green I PCR Master Mix (Qiagen) and 5 μmol/l of gene-specific primer pairs. The following primer pairs for *fbpB* (FW: 5′- GGGACGCCGATTGATGAT-3′; RV: 5′- CGCTCTGGAACTGAACCTTG-3′); *cysD* (FW: 5′- AGTCATCGCCGAAACTGC-3′; RV: 5′- TTGCCATCGTCGACGGAA-3′); and *rrs* (FW: 5′- AGGCAGCAGTGGGGAATA-3′; RV: 5′- CTACCGTCAATCCGAGAGAA-3′) were used for amplification. The reactions were run on a Rotor-gene 6000-series sequence detector (Corbett Life Science, Sydney, Australia) in triplicates per primer pair. Relative expression levels were determined using the comparative threshold cycle method of Livak and Schmittgen^53^ with the *rrs* sequence, encoding the 16S ribosomal RNA of *M. tuberculosis,* as the reference gene.^54^

### Vaccination and infection of mice

Female C57BL/6, BALB/c or Q(s) mice (6–8 weeks of age) were purchased from the Animal Resources Centre (Perth, Australia). Mice were maintained in specific pathogen-free condition and experiments were performed with the approval of the University of Sydney Animal Care and Ethics Committee (approval number K75/9-2012/3/5846). Animals were randomly assigned to experimental groups, experiments were not blinded. Number of animals required per group was based on the ability to detect a 40% difference between groups, a significance of *P*<0.05 and 80% power to reject the null hypothesis given the alternative hypothesis is true.

For protection experiments, 6-week-old female C57BL/6 were vaccinated subcutaneously (s.c.) at the base of the tail either once with 5×10^5^ CFU of BCG Pasteur (200 μl in PBS), or three times at 2 weeks interval with 3 μg of recombinant protein formulated in monophosphoryl lipid A/dimethyl dioctadecyl ammonium bromide (MPL/DDA, 25 μg/250 μg per dose, respectively, 200 μl total volume).^52^ Six weeks after the final vaccination, the mice were challenged with *M. tuberculosis* H37Rv via aerosol route using a Middlebrook airborne infection apparatus (Glas-Col) with an infective dose of ~100 viable bacilli per lung. Four weeks later the lung and spleen were collected, homogenised and plated after serial dilutions on supplemented Middlebrook 7H11 agar plates. Colonies forming units (CFU) were determined ~3 weeks later and expressed as Log_10_ CFU.

### Immunogenicity assays

Splenocytes and mediastinal lymph nodes were prepared from vaccinated or infected mice and passaged through a cell strainer (BD). Whole lung was perfused post-mortem through the heart with PBS/heparin, and lung cells were dissociated with Collagenase I (0.1 mg ml^−1^; Worthington, Lakewood, NJ, USA) and DNase (10 U ml^−1^; Worthington) using a GentleMACS Dissociator (Miltenyi Biotec, Bergisch Gladbach, Germany) as per the manufacturer’s instructions. Cells were resuspended in buffered ammonium sulfate (ACK buffer; 0.1 mM EDTA (Sigma), 10 mM KHCO_3_ (Sigma), 150 mM NH_4_Cl (Sigma)) to lyse erythrocytes and then washed and resuspended in RPMI 1640 (Life Technologies) supplemented with 10% heat-inactivated fetal bovine serum (Scientifix, Cheltenham, Australia), 50 μM 2-β-mercaptoethanol (Sigma), and 100 U ml^−1^ Penicillin/Streptomycin (Sigma). Antigen-specific IFN-γ producing cells were detected by ELISPOT assay as described previously.^55^ Antigens for stimulation (Ag85B, CysD, CysVac2) were used at a concentration of 10 μg ml^−1^. For Cytokine ELISAs, cells were stimulated with antigen and supernatants collected after 72 h, and IFN-γ was detected as described previously.^55^

### Intracellular cytokine staining and flow cytometry

For intracellular cytokine staining, cells were stimulated for 3–4 h in the presence of the CysVac2 fusion protein (10 μg ml^−1^) or *M. tuberculosis* CFP (10 μg ml^−1^) and then for up to 12 h with brefeldin A (10 μg ml^−1^). Two million cells were incubated with 1.25 μg ml^−1^ anti-CD32/CD16 (eBioscience, San Diego, CA, USA) in FACS wash buffer (PBS/2% FCS/0.1%) for 30 min to block Fc receptors, then washed and incubated for 30 min with a mix of fluorescent Abs: anti-CD3-PerCPCy5.5, anti-CD4-Alexafluor 700, anti-CD8-allophycocyanin (APC)-Cy7, and anti-CD44- fluorescein isothiocyanate (BD), Fixable Blue Dead Cell Stain (Life Technologies) was added to the Ab mix at the dilution recommended by the manufacturer to allow dead-cell discrimination. Cells were then fixed and permeabilised using the BD Cytofix/Cytoperm kit according to the manufacturer’s protocol. Intracellular staining was performed using the following antibodies: anti-IFN-γ-phycoerythrin (PE)-Cy7 (eBioscience), anti-TNF-APC (Biolegend, San Diego, CA, USA), anti-IL-2-PE (BD) and anti-IL-17A-Pacific Blue (Biolegend). Samples were acquired on a BD LSR-Fortessa (BD) and analysed using FlowJo analysis software (Treestar, Macintosh Version 9.8, Ashland, OR, USA) using the gating strategy described in the Supplementary Figure 1. A Boolean combination of gates was used to calculate the frequency of single-, double- and triple-positive CD3^+^CD4^+^ cell subsets.

### Histology

The middle right lobe of each infected mouse was perfused with a 10% buffered formalin solution, paraffin embedded and stained with haematoxylin and eosin. Slides were observed with LeicaDM microscope (Leica Microsystems, North Ryde, Australia) with a magnification of ×10 and acquired as a mosaic.

### Statistical analysis

The significance of differences between experimental groups was evaluated by one- or two-way analysis of variance, with pairwise comparison of multi-grouped data sets achieved using Tukey or Dunnet *post hoc* test.
