# Supplementary figures and images for: Mycobacterium tuberculosis components expressed during chronic infection of the lung contribute to long-term control of pulmonary tuberculosis in mice
Source: NPJ Vaccines. 2016 Sep 15;1:16012–. doi: 10.1038/npjvaccines.2016.12 (PMC5707878; doi:10.1038/npjvaccines.2016.12)

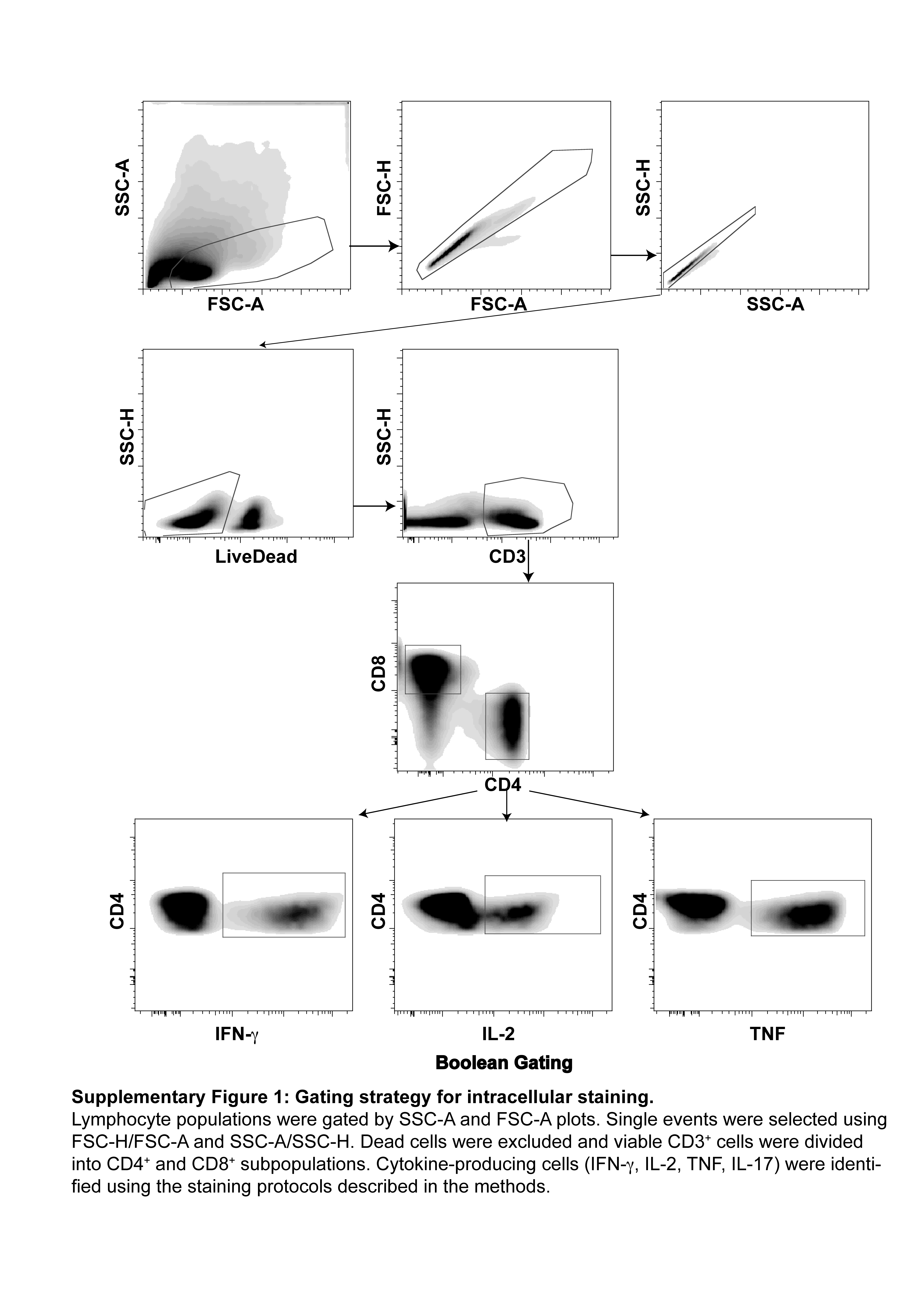

Supplement: Supplementary Figure 1 [file npjvaccines201612-s1.tiff]

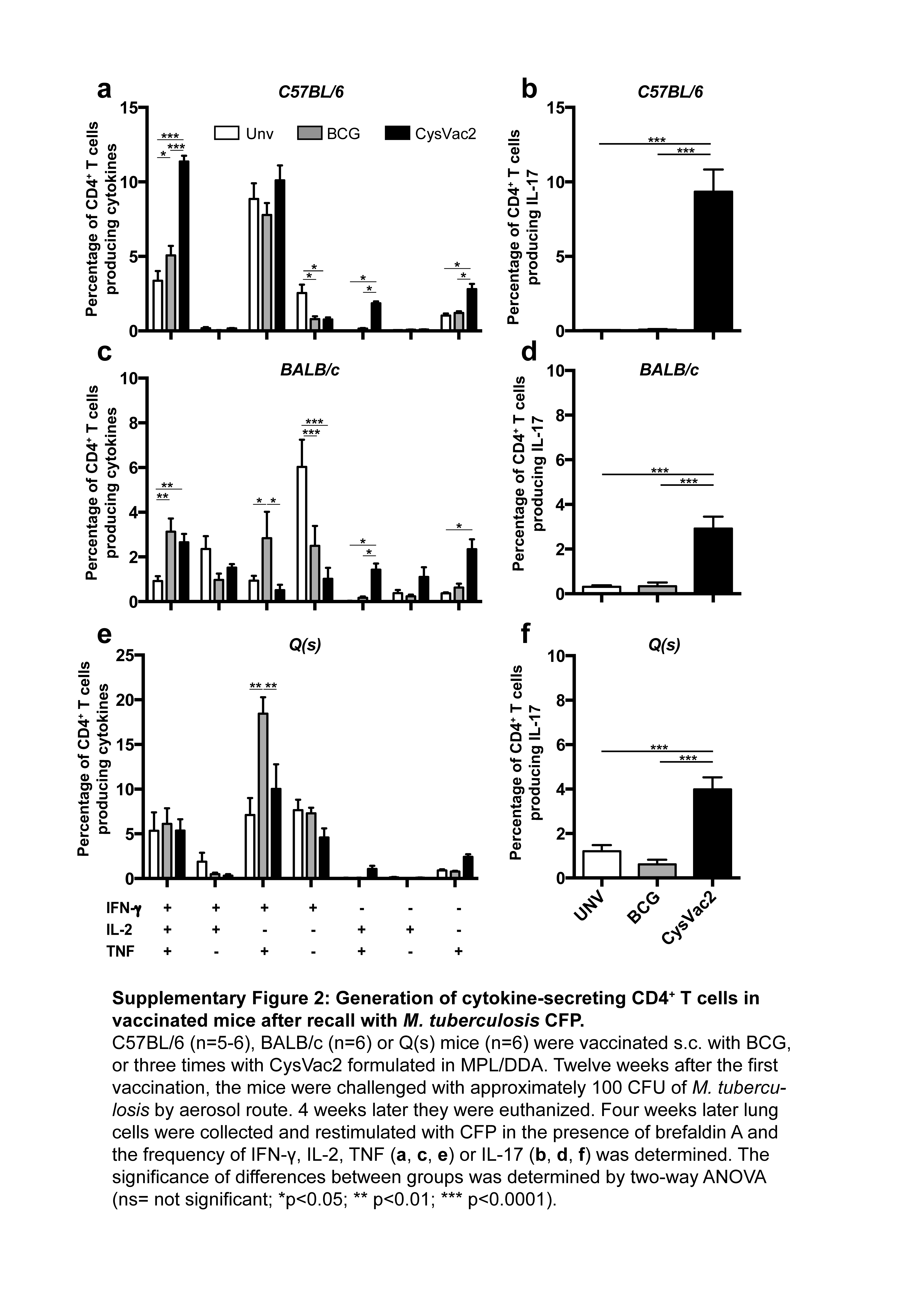

Supplement: Supplementary Figure 2 [file npjvaccines201612-s2.tiff]
